# Pattern of failure in prostate cancer previously treated with radical prostatectomy and post-operative radiotherapy: a secondary analysis of two prospective studies using novel molecular imaging techniques

**DOI:** 10.1186/s13014-020-01733-x

**Published:** 2021-02-10

**Authors:** Lindsay S. Rowe, Stephanie Harmon, Adam Horn, Uma Shankavaram, Soumyajit Roy, Holly Ning, Liza Lindenberg, Esther Mena, Deborah E. Citrin, Peter Choyke, Baris Turkbey

**Affiliations:** 1 Radiation Oncology Branch, National Cancer Institute, 10 Center Drive Magnuson Clinical Center, Room B2-3500, Bethesda, MD 20892 USA; 2 Department of Radiation Oncology, Cross Cancer Institute, 11560 University Avenue, Edmonton, AB T6G 1Z2 Canada; 3 Clinical Research Directorate, Frederick National Laboratory for Cancer Research, 10 Center Drive Magnuson Clinical Center, Room B3B69F, Bethesda, MD 20892 USA; 4 Walter Reed National Military Medical Center, Bethesda, MD 8901 Rockville Pike USA; 5 Radiation Oncology Branch, National Cancer Institute, 10 Center Drive Magnuson Clinical Center, Room 1002, Bethesda, MD 20892 USA; 6 Molecular Imaging Program, National Cancer Institute, 10 Center Drive Magnuson Clinical Center, Room B3B69F, Bethesda, MD 20892 USA

**Keywords:** Prostate cancer, Prostate-specific membrane antigen (PSMA), PET/CT, F-18, Biochemical recurrence, Radiation therapy, Prostatectomy

## Abstract

**Background:**

Prostate Membrane Specific Antigen (PSMA) positron emission tomography (PET) and multiparametric MRI (mpMRI) have shown high accuracy in identifying recurrent lesions after definitive treatment in prostate cancer (PCa). In this study, we aimed to outline patterns of failure in a group of post-prostatectomy patients who received adjuvant or salvage radiation therapy (PORT) and subsequently experienced biochemical recurrence, using ^18^F-PSMA PET/CT and mpMRI.

**Methods:**

PCa patients with biochemical failure post-prostatectomy, and no evident site of recurrence on conventional imaging, were enrolled on two prospective trials of first and second generation ^18^F-PSMA PET agents (^18^F-DCFBC and ^18^F-DCFPyL) in combination with MRI between October 2014 and December 2018. The primary aim of our study is to characterize these lesions with respect to their location relative to previous PORT field and received dose.

**Results:**

A total of 34 participants underwent ^18^F-PSMA PET imaging for biochemical recurrence after radical prostatectomy and PORT, with 32/34 found to have ^18^F-PSMA avid lesions. On ^18^F-PSMA, 17/32 patients (53.1%) had metastatic disease, 8/32 (25.0%) patients had locoregional recurrences, and 7/32 (21.9%) had local failure in the prostate fossa. On further exploration, we noted 6/7 (86%) of prostate fossa recurrences were in-field and were encompassed by 100% isodose lines, receiving 64.8–72 Gy. One patient had marginal failure encompassed by the 49 Gy isodose.

**Conclusions:**

^18^F-PSMA PET imaging demonstrates promise in identifying occult PCa recurrence after PORT. Although distant recurrence was the predominant pattern of failure, in-field recurrence was noted in approximately 1/5th of patients. This should be considered in tailoring radiotherapy practice after prostatectomy.

*Trial registration* www.clinicaltrials.gov, NCT02190279 and NCT03181867. Registered July 12, 2014, https://clinicaltrials.gov/ct2/show/NCT02190279 and June 8 2017, https://clinicaltrials.gov/ct2/show/NCT03181867.

## Background

Novel imaging modalities, such as Prostate Membrane Specific Antigen (PSMA) targeted positron emission tomography (PET) and multiparametric magnetic resonance imaging (mpMRI), have ushered in a new era for defining patterns of disease recurrence and metastatic spread in patients with prostate cancer (PCa). PSMA PET has had success in determining PCa patterns of spread for initial staging and at the time of biochemical recurrence in patients who otherwise have no identifiable disease on standard imaging techniques, such as computed tomography (CT) and bone scan.

Studies addressing PCa patterns of failure post-prostatectomy with PSMA targeted PET and mpMRI have found that in patients restaged prior to salvage radiation 10–30% have locally recurrent disease in the prostate fossa, and 70% of patients have alterations in TNM stage [1–4]. These insights into common sites of failure are highly valuable in correctly stratifying patients by stage of disease at the time of biochemical recurrence. Additionally, even at low PSA levels, PSMA targeted imaging demonstrates significant impact on salvage radiation planning, with a portion of patients having PSMA positive lesions outside of consensus radiation volumes despite being candidates for therapy [5].

One related research question with important therapeutic implication is the definition of the patterns of failure after post-prostatectomy radiation therapy (PORT). Although the patterns of recurrence after prostatectomy and before PORT are important for identifying radiation targets for salvage, understanding patterns of failure after PORT may be additionally insightful. Theoretically the use of localized radiation to the prostate fossa should sterilize microscopic disease in the irradiated area, with patterns of failure expected to shift to nodal and distant recurrences. Evidence of recurrences outside of the field could argue for field size changes or systemic therapy, whereas failure inside the treatment field after PORT may argue for dose escalation or other treatment intensification. Therefore, this study aims to outline patterns of failure in a group of post-prostatectomy patients who received adjuvant or salvage radiation therapy (PORT) and subsequently experienced biochemical recurrence.

## Methods

### Study population

All study participants were enrolled in one of two institutional review board approved prospective, single institutional clinical trials that accrued patients between October 2014 and December 2018 with the primary aim of assessing the ability of ^18^F-PSMA PET to detect sites of recurrent PCa. Written informed consent was obtained from all subjects.

The first study of N-[N-[(S)-1,3-dicarboxypropyl]carbamoyl]-4-F-fluorobenzyl-l-cysteine (^18^F-DCFBC) PET/CT accrued participants in three arms with varying eligibility and has been published previously [6]. The second study enrolled participants on a biochemical recurrence arm of 2-(3-{1-carboxy-5-[(6-[^18^F]fluoro-pyridine-3-carbonyl)-amino]-pentyl}-ureido)-pentanedioic acid (^18^F-DCFPyL) PET/CT (NCT03181867). Together ^18^F-DCFBC and ^18^F-DCFPyL are referred to as ^18^F-PSMA, and all patients received mpMRI of the prostate fossa with their ^18^F-PSMA PET/CT scans. Inclusion criteria for both protocols were biochemical recurrence after primary therapy for adenocarcinoma of the prostate, PSA of ≥ 0.2 ng/ml, and no site of recurrence visible on standard imaging (CT of the abdomen and pelvis, and planar bone scan). The current manuscript entails a secondary analysis of a subgroup of these participants who experienced biochemical recurrence after radical prostatectomy and adjuvant or salvage radiation therapy (PORT) with no detectable lesions on conventional anatomical imaging.

### mpMRI, ^18^F-DCFBC and ^18^F-DCFPyL PET/CT imaging protocols

Details of image acquisition and protocol work flow for mpMRI and ^18^F-DCFBC PET/CT have been discussed previously and remained unchanged with ^18^F-DCFPyL PET/CT [6, 7]. Two board certified nuclear medicine physicians prospectively read all data sets independently, resolving any disagreements by consensus, and documented consensus lesions as key images. Any abnormal focus of ^18^F-PSMA PET/CT uptake higher than the surrounding background and not associated with physiological uptake was considered positive for recurrent prostate cancer, and each was classified as local recurrence, regional lymph node metastases, or distant metastatic sites.

### ^18^F-PSMA PET/CT image fusion and radiation therapy dosimetry

**Fig. 1:**
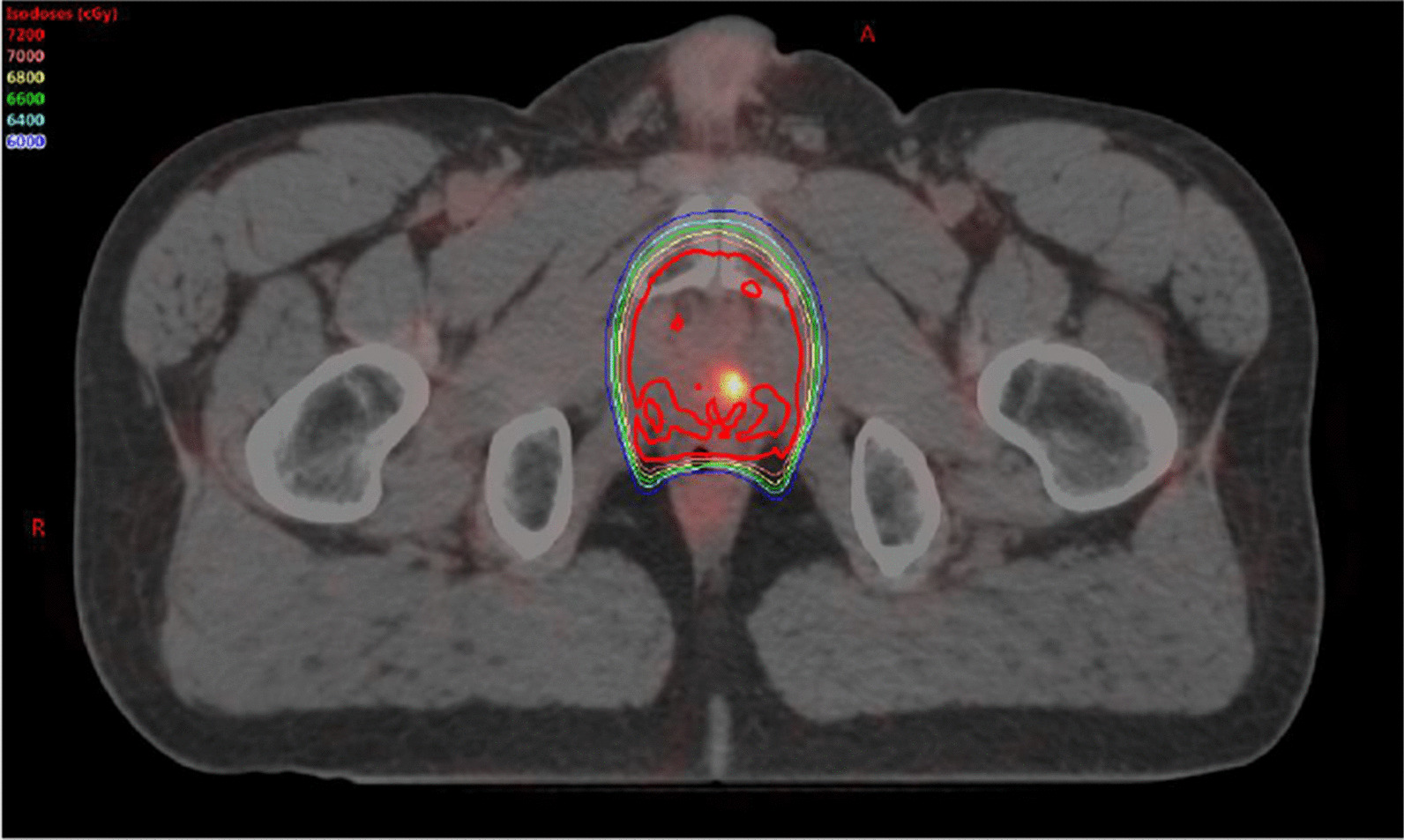
58-year-old male with a serum PSA of 3.09 ng/ml after radical prostatectomy and salvage radiotherapy with ADT. ^18^F-PSMA-PET image overlaid with radiation treatment dosimetry. The patient received a prescribed dose of 70.2 Gy

^18^F-PSMA PET/CT and mpMRI images were imported into Eclipse Treatment Planning System (Varian, Stanford, CA) for target delineation. ^18^F-PSMA PET/CT avid lesions were contoured by a board certified nuclear medicine physician, based on previously defined consensus key images, on the corresponding ^18^F-PSMA PET/CT scans while blinded to prior radiation therapy dosimetry. Patients were classified into three groups based on the location of ^18^F-PSMA PET/CT avid lesions: local recurrence only (disease only within the region of the prostate fossa), locoregional recurrence (disease limited to the pelvic lymph nodes with or without disease in the prostate fossa), or metastatic disease (at least one lesion outside of the pelvis with or without disease in the prostate fossa or regional lymph nodes) [8, 9]. Metastatic disease was further subdivided into patients with distant metastatic lesions with or without regional pelvic nodal disease. Original radiation therapy plans and treatment summaries for patients who had local or locoregional recurrences were obtained for individual patients. When available, previous radiation plans were uploaded on the Eclipse planning system and ^18^F-PSMA PET/CT lesion contours were then fused to previous radiation therapy dose distributions, and radiation doses to the ^18^F-PSMA PET/CT avid areas were defined (Fig. 1). In patients who did not have DICOM images available, dose distributions from printed plans were used, and if possible, it was determined if areas of recurrence were in the treatment volume based on anatomic landmarks by a board certified radiation oncologist. The concordance of mpMRI and ^18^F-PSMA PET/CT avid areas with previous radiation plans were evaluated descriptively both on a per lesion and per patient basis.

### Statistical analysis

Patient demographics were reported descriptively. Clinical variables were analyzed to assess their impact on the pattern of relapse (local recurrence, locoregional recurrence, or distant metastases). Analyzed continuous variables included age at diagnosis, post-prostatectomy PSA, PSA at recurrence, PSA level at the time of ^18^F-PSMA PET, time to biochemical failure, and radiation dose to the prostate fossa. Discrete variables analyzed included Gleason grade group (1–5), NCCN risk group, surgical margin status, seminal vesicle invasion, extracapsular extension, pathologic lymph node status, and prior exposure to ADT. All statistical analyses were performed using R software environment [10]. Multinomial logistic regression was used for both univariate and multivariate regression models with site of recurrence (local, locoregional, distant) as the dependent variable. Univariate and multivariate regression models were used to evaluate factors associated with site of recurrence at the time of ^18^F-PSMA PET. A tidy function was used to get component level statistics from the multinomial models. To prevent overfitting of the models, all variables were tested for multicollinearity, and extreme outliers before model fitting. Regression analyses were performed only after confirmation that the model assumptions were met using metastasis as reference. For univariate analysis, the main effects of each of the 13 variables was independently tested using separate models. Unified estimates including odds ratio and 95% confidence intervals (CI) were calculated. A *p* value was computed by Chi square for categorical, and Student’s t test for continuous variables, with a cutoff of ≤ 0.05 for statistical significance. The aptness of the multinomial logistic regression model was evaluated by Akaike Information Criterion (AIC).

## Results

A total of 120 participants underwent ^18^F-PSMA PET imaging for biochemical recurrence after radical prostatectomy; among them 34 (25%) received PORT prior to ^18^F-PSMA PET scan, and 32/34 (94.1%) patients had detectable lesions on ^18^F-PSMA imaging. For this cohort of 32 patients, the median age at prostate cancer diagnosis was 56.8 years, and 15 out of 32 patients (46.9%) received ADT prior to ^18^F-PSMA PET/CT. Median time to biochemical failure post-prostatectomy was 30.1 months (range 1.1–113.9). Median time between PORT and ^18^F-PSMA PET/CT was 44.9 months (range 14.5–168.2). Median PSA was 2.42 ng/ml (range 0.38–22.37) at the time of ^18^F-PSMA PET/CT.

Radiation delivery for the 32 patients ranged from December 2004 to June 2017 and involved both 3D conformal and IMRT. Eight patients received adjuvant radiation, and 24 received radiation due to a detectable PSA. Median time to biochemical recurrence after adjuvant PORT was 33.1 months (range 3.0–106.6), and time to biochemical recurrence prior to salvage radiation was 15.9 months (range 1.1–94.1). All patients received radiation to the prostate fossa with a median dose of 68.4 Gy (range 64.8–72); 6/32 (18.7%) patients received elective pelvic nodal radiation to 45 Gy. Of the six patients who received regional nodal radiation as a component of PORT, one patient failed in the pelvic lymph nodes, one failed in the prostate fossa, and all others were distant metastatic recurrences (4/6).

**Fig. 2:**
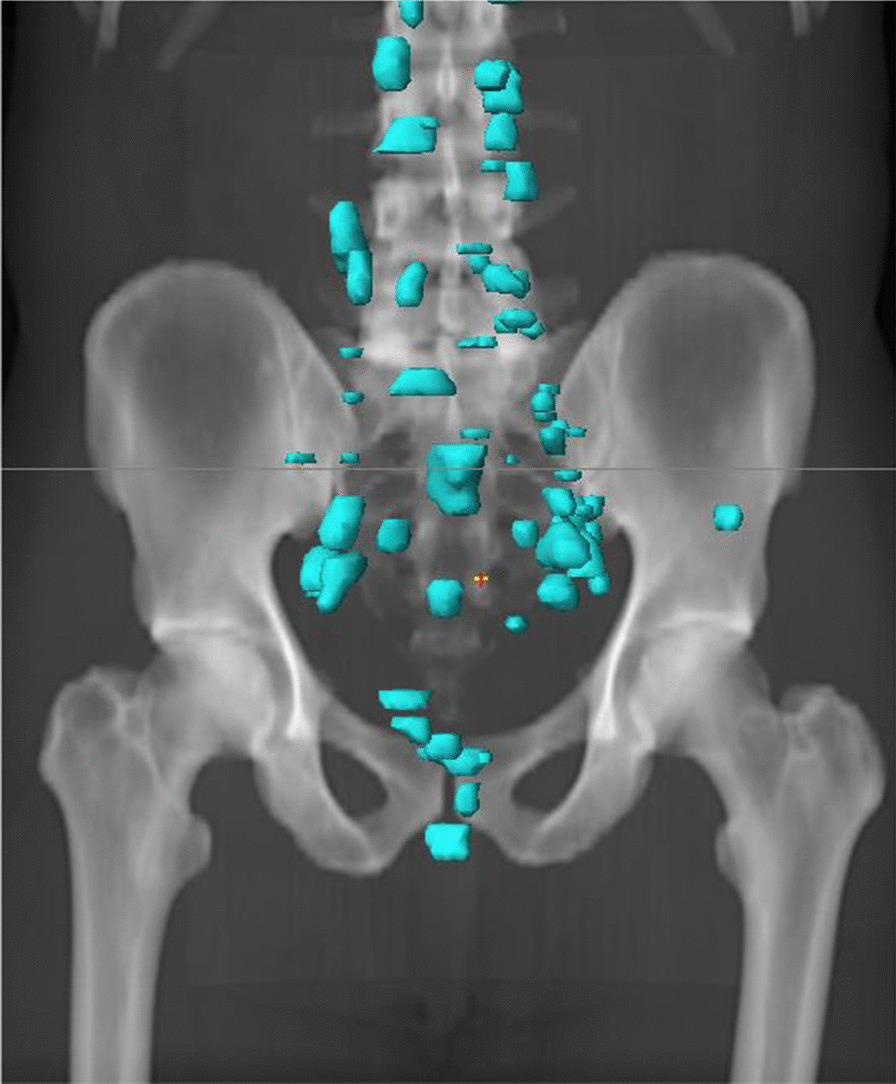
Distribution of ^18^F-PSMA PET/CT avid lesions in the abdomen and pelvis (pulmonary and supraclavicular metastases not shown) for the 32 patients included in the analysis

None of the patients had PSMA PET/CT prior to PORT. Three of the 32 patients had MRI imaging of the prostate fossa prior to PORT, one with a detectable lesion, and two without. In the patient with a pre-PORT detectable lesion, standard salvage radiation fields were used, and no radiation dose escalation was delivered to the MRI detectable lesion (Table 2, Patient 5). Of the 32 patients, only 2 received their radiation at the ^18^F-PSMA institution. Patient characteristics for the 32 are presented in Table 1, and locations of ^18^F-PSMA PET/CT avidity are presented in Fig. 2.

**Table 1:** Patient characteristics for all patients, and subgroups of local, locoregional, and metastatic recurrence

	All patients^a^		Local recurrence^b^		Locoregional recurrence^c^		Metastatic disease^d^			
							Regional nodal and metastatic lesions		Metastatic lesions alone	
	n = 32	%	n = 7	%	n = 8	%	n = 13	%	n = 4	%
Median age at diagnosis	56.5 (range 52.5–73.7)		55.0 (range 53.0–61.5)		57.2 (range 53.1–69.4)		59.2 (range 53.0–73.7)		58.6 (range 52.5–66.8)	
Tumor stage										
T2	14	43.8%	5	62.5%	3	37.5%	3	23.1%	3	75.0%
T3a	8	25.0%	1	12.5%	4	50.0%	3	23.1%	0	0%
T3b	9	28.1%	1	12.5%	1	12.5%	6	46.2%	1	25.0%
T4	1	3.1%	0	0.0%	0	0.0%	1	7.7%	0	0%
Nodal stage										
N0	26	81.3%	6	75.0%	7	87.5%	10	76.9%	3	75.0%
N1	1	3.1%	0	0.0%	0	0.0%	1	7.7%	0	0%
Nx	5	15.6%	1	12.5%	1	12.5%	2	15.4%	1	25.0%
Gleason score										
6	2	6.3%	2	25.0%	0	0.0%	0	0%	0	0%
7 (3 + 4)	8	25.0%	2	25.0%	3	37.5%	1	7.7%	2	50.0%
7 (4 + 3)	11	34.4%	2	25.0%	1	12.5%	8	61.5%	0	0%
8	8	25.0%	1	12.5%	2	25.0%	4	30.8%	1	25.0%
9	3	9.4%	0	0.0%	2	25.0%	0	0%	1	25.0%
Positive surgical margins	13	40.6%	3	37.5%	3	37.5%	7	53.8%	0	0%
Median time to biochemical failure	30.1 mo (1.1–113.9)		30.5 mo (1.4–86.3)		27 mo (2.7–113.9)		15.8 mo (1.1–82.0)		49.9 mo (1.4–94.3)	
Median PSA at ^18^F-PSMA PET	2.4 ng/mL (0.38–22.37)		1.3 ng/ml (0.5–3.3)		1.6 ng/ml (0.4–8.9)		4.8 ng/ml (1.3–22.4)		5.4 ng/ml (2.0–7.9)	

^a^All 32 patients included in the analysis of those who received PORT after prostatectomy prior to mpMRI and PSMA PET/CT imaging as per inclusion criteria

^b^Subgroup of the 32 patients with local recurrence alone on mpMRI and PSMA PET/CT

^c^Subgroup of the 32 patients who were found to have regional nodal recurrence (± local) on mpMRI and PSMA PET/CT

^d^Subgroup of the 32 patients who were found to have metastatic (± local or regional nodal) disease on mpMRI and PSMA PET/CT

Seventeen of 32 patients (53.1%) were found to have metastatic disease at the time of ^18^F-PSMA PET/CT scan, with a total of 78 lesions. For these 17 patients, median PSA at the time of ^18^F-PSMA PET was 4.94 ng/mL (range 1.31–22.37) and the median time to biochemical recurrence after prostatectomy was 42.9 months (range 1.1–94.3). None of the patients were found to have a local recurrence in the prostate fossa. Regarding the number of metastatic sites, four patients had a solitary site of metastasis, 5 patients had 2–3 sites of disease, and one patient had 5 sites of disease. The remaining patients had high metastatic burden with ≥ 7 sites.

Of these 17 patients with metastatic disease, 13 patients had regional nodal disease in additional to their distant sites of metastases, and 4 patients had distant metastases alone. The regional lymph nodes involved included the obturator, internal iliac, external iliac, common iliac, and presacral nodes. Sites of distant metastases included paraaortic, aortocaval, retrocaval, hilar and supraclavicular lymph nodes, in addition to bone and lung, Demographics of these participants are presented in Table 1.

Eight of 32 (25.0%) patients presented with locoregional recurrences, with 16 lesions identified as positive lymph nodes in the peri-rectal, obturator, internal and external iliac regions. None of these patients were found to have a local recurrence in the prostate fossa in addition to their regional nodal disease. Median PSA at the time of ^18^F-PSMA scan was 1.60 ng/mL (range 0.38–8.92). Patients’ time to biochemical recurrence after prostatectomy was 27.0 months (range 2.7–113.9). All patients had received radiation to the prostate fossa, but only one of these patients with regional nodal recurrence had received pelvic nodal radiation to 45 Gy. Demographics of these participants are presented in Table 1.

**Fig. 3:**
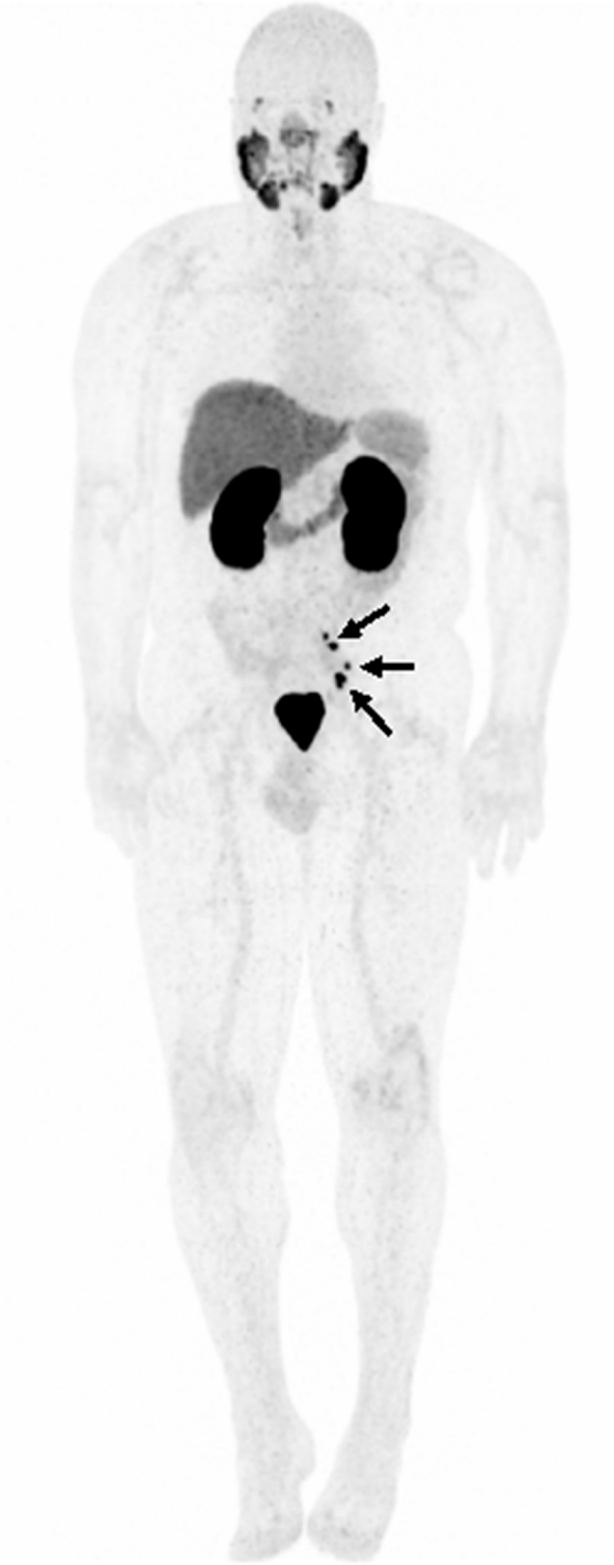
57-year-old male with a serum PSA of 3.14 ng/ml after radical prostatectomy and salvage radiation and ADT. ^18^F-DCFPYL PET/CT shows focal radiotracer uptake within the left iliac chain normal size lymph nodes (arrows)

**Fig. 4:**
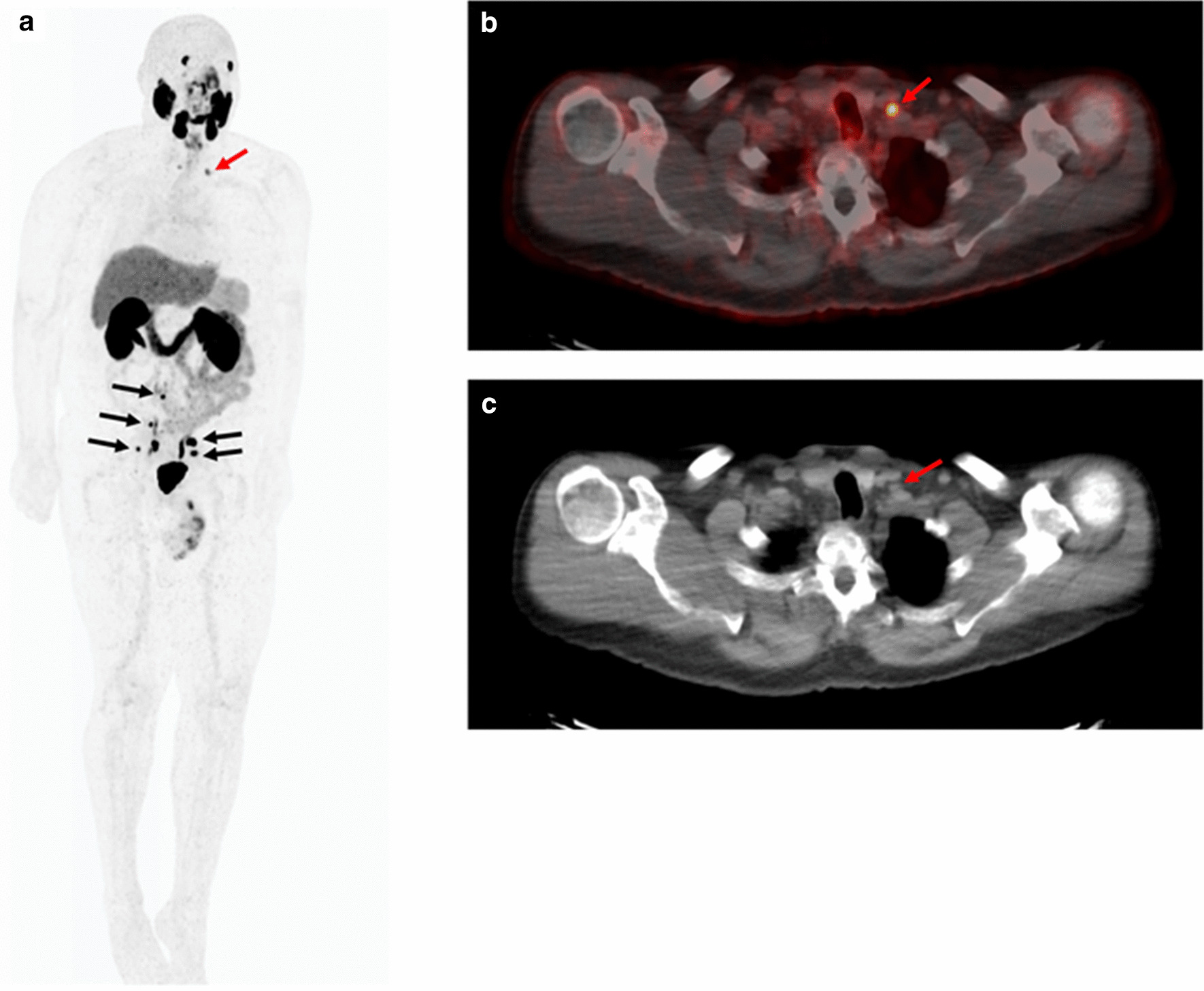
66-year-old male with serum PSA of 4.83 ng/ml after radical prostatectomy and salvage radiation therapy. ^18^F-DCFPYL PET/CT shows focal radiotracer uptake within bilateral pelvic lymph nodes (black arrows) (**a**) and in a normal sized supraclavicular lymph node (red arrows) (**a**–**c**)

**Fig. 5:**
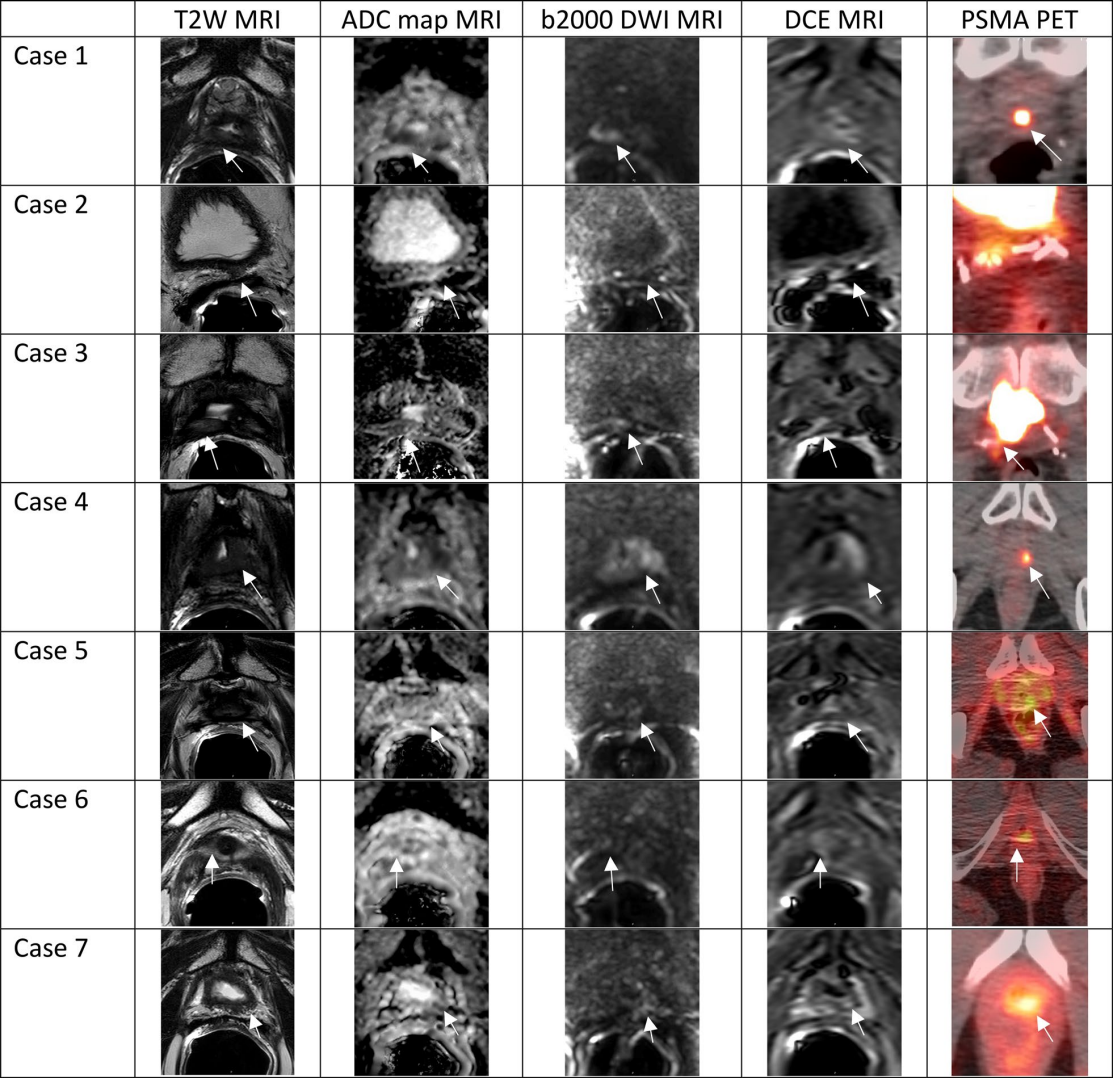
Axial MRI and ^18^F-PSMA PET/CT images of each of the seven patients with isolated local recurrence. White arrows point to areas of interest

Interestingly, 21.9% (7/32) of patients presented with local recurrences in the prostate fossa alone, despite receiving prior radiation therapy to the area. Median PSA at the time of ^18^F-PSMA scan was 1.31 ng/mL (range 0.54–3.31). Each patient had a single lesion with an average maximum diameter of 1.14 cm (range 0.4–2.3) as measured on mpMRI, with two lesions pathologically confirmed as recurrent prostate cancer. The remaining 5 lesions could not be visualized by ultrasound guidance for biopsy. ^18^F-PSMA PET/CT and mpMRI lesions were correlated in 6/7 patients, whereas one patient (Case 2, Fig. 5) was found to have a lesion on mpMRI that was not correlated to PET/CT uptake. Overall six patients (85.7%) recurred within the 100% radiation isodose, having been prescribed between 66.6 and 72 Gy while one (14.3%) patient recurred locally in the prostate fossa, but outside of the radiation target, the lesion having received 49 Gy of marginal dose. Patient characteristics and radiation treatment summaries for patients with post-radiotherapy local recurrence is presented in Table 2, and their location in relation to the radiation field and target volume have been demonstrated in Figs. 3, 4, and 5.

**Table 2:** Clinical and treatment parameters for patients with isolated local recurrence

	Patient 1	Patient 2	Patient 3	Patient 4	Patient 5	Patient 6	Patient 7
Age at diagnosis (years)	56	57	54	53	55	55	62
NCCN risk group	High	Intermediate	High	Intermediate	Intermediate	Intermediate	Intermediate
Preoperative PSA (ng/ml)	4.5	4	4.9	5.7	4.20	10.00	6.80
Gleason score	7 (4 + 3)	7 (4 + 3)	8 (4 + 4)	7 (3 + 4)	6 (3 + 3)	6 (3 + 3)	7 (3 + 4)
Pathologic T-stage	T3b	T2c	T3a	T2	T2c	T2c	T2c
Pathologic N-stage	N0	N0	N0	N0	Nx	N0 (0/5)	N0 (0/8)
Extraprostatic extension	Present	None	Present	None	Negative	Negative	Negative
Seminal vesicle invasion	Present	None	None	None	Negative	Negative	Negative
Operative margins	Negative	Negative	Positive	Negative	Negative	Positive	Positive
Post-operative PSA	Undetectable	Undetectable	0.2	Undetectable	Detectable	0	2.23
Time to biochemical failure (months)	30.5	86.3	3.2	72.5	3.53	45.47	1.4
Adjuvant or Salvage Radiation	Adjuvant	Salvage	Salvage	Salvage	Salvage	Salvage	Salvage
Radiation field	Prostate fossa	Prostate fossa	Prostate fossa	Prostate fossa	Prostate Fossa	Prostate fossa	Prostate fossa + LN
Radiation dose prescription	7200 cGy	7020 cGy	6840 cGy	7200 cGy	7020 cGy	6660 cGy	6840 cGy
Recurrence within 100% isodose	Yes	Yes	Yes	No (outside of contour)	Yes	Yes	Yes
Dose to recurrence lesion	N/A	7020 cGy	6840 cGy	4900 cGy	7020 cGy	6660 cGy	N/A
PSA at ^18^F-PSMA PET/CT (ng/ml)	0.54	0.83	1.01	1.39	3.09	1.31	3.31

Exploratory analysis of factors associated with location of recurrence (local, locoregional, and metastatic) on ^18^F-PSMA PET imaging after prostatectomy and PORT demonstrated Gleason grade group, risk category, LN status at prostatectomy, and PSA at the time of ^18^F-PSMA PET as significant in univariate analysis (Additional file 1: Table 1). Multivariate analysis was performed on selected features found to be significant (*p* value ≤ 0.05) in univariate analysis (Additional file 3: Table 3). Gleason grade group, LN status at prostatectomy, and PSA at the time of ^18^F-PSMA PET were found to be significant in multivariate analysis. However, on component level statistics only PSA at PSMA-PET was significant for metastases, and Gleason Grade Group for local recurrence (Additional file 2: Table 2).

## Discussion

The use of novel prostate cancer imaging techniques is providing new insight into staging and treatment planning for definitive and salvage therapies for this disease. The current study provides a unique perspective on the patterns of recurrence after PORT and their relationship to prior radiation fields. To our knowledge this is the largest cohort of post-prostatectomy patients with PORT assessing patterns of failure with ^18^F-PSMA PET/CT and mpMRI imaging. These data support previous ^68^Ga-PSMA PET findings that in patients with biochemical recurrence, and negative conventional imaging, metastatic disease is a predominant pattern of failure in patients after PORT [11, 12]. Using ^18^F-PSMA based imaging techniques and mpMRI, the present study identified 20% of patients receiving PORT failed inside the treatment field, a higher rate than previously documented. This finding brings into question if intensification of therapy, such as dose escalation or the addition of systemic agents, may have efficacy in reducing local recurrence in the radiation field.

The literature uniquely addressing PSMA targeted imaging techniques for biochemical recurrence after prostatectomy and PORT is limited. One notable study by Byrne et al. (2017) assessed patterns of recurrence after PORT in 81 men who underwent ^68^Ga-PSMA-PET/CT imaging [11]. They found a rate of 4% local recurrence in the prostate fossa after irradiation, with 28% regional nodal recurrences. The authors also separated participants based on prostate fossa alone, or prostate fossa with regional nodal irradiation, and found rates of 4% and 6% in field recurrences respectively. While rates of nodal detection are similar between our study groups, this is not unexpected, as PSMA PET imaging has been highlighted to improve nodal detection not otherwise identified on conventional imaging [13]. However, the rate of 4% local recurrence is lower than in the current patient cohort and may be accounted for by the either the addition of mpMRI or a ^18^F-PSMA agent. Rates of local recurrence may be underestimated by ^68^Ga-PSMA PET/CT imaging alone which relies on the lower spatial resolution of non-contrast CT scans to provide anatomic localization. PSMA PET/CT can also be limited by urinary activity obscuring recurrence near the anastomosis, a situation which may be resolved with the improved spatial resolution of mpMRI. mpMRI imaging has demonstrated ability to improve the detection of local recurrence in patients with biochemical recurrence post-prostatectomy. A cohort of post-prostatectomy patients with biochemical recurrence, but no previous PORT, demonstrated significant improvement in the detection of local recurrences with the addition of mpMRI to PET/CT [14]. In 26 patients imaged with ^68^Ga-PSMA-PET/CT, rates of detecting local recurrence improved from 14.5% with PET/CT alone to 26.3% with the addition of mpMRI [14]. A recent retrospective cohort of 251 patients undergoing ^18^F-PSMA-1007 PET/CT imaging assessed patterns of failure post-prostatectomy, and 43.8% had received salvage radiation therapy prior to imaging [15]. In this population of prostatectomy patients with or without PORT, rates of local recurrence ranged from 18.5 to 31% depending on PSA at the time of ^18^F-PSMA-1007 PET/CT, with an increase in rates of nodal and metastatic disease with increased PSA [15]. Similarly, in the current study patients presenting with local recurrence had a median PSA 1.31 ng/ml, versus 1.6 ng/ml and 4.49 ng/ml for nodal and metastatic disease.

With the benefit of novel imaging, mpMRI and ^18^F-PSMA PET, this data provides important insight into patterns of failure post-prostatectomy after PORT. While PORT is effective in salvaging many patients post-prostatectomy, those with biochemical recurrence and negative conventional imaging after PORT often move on to systemic therapies with the assumption that they harbor micrometastatic disease. However, these data suggest that a clinically significant proportion of these patients still harbor locally recurrent disease. Although these data are only hypothesis generating, they suggest utility in including an assessment of patterns of failure in future studies that aim to reduce the risk of recurrence after PORT with dose escalation, modified treatment volume, or the use of systemic therapies. This is supported by retrospective evidence that suggests that dose escalation to the prostate bed increases biochemical relapse free survival, and disease free survival [16–18]. In this study one patient experienced a recurrence at the PORT field edge. Similar rates of 7–12% edge of treatment field lesions have been noted by others [5, 19], and continued analysis of patterns of failure in larger patient cohorts may help better inform radiation volume delineation and already trials using image guided radiation planning are underway [20, 21]. Additionally, one of the findings in this data was that no patients with nodal or distant metastatic disease also had a recurrent lesion in the prostate fossa. This may be related to the small sample size, however, it may also signify different biologic processes between recurrence patterns. These isolated local recurrences were also identified at a range of PSA levels (0.5–3.3 ng/ml). In future studies, it would be of interest of evaluate if these observed patterns of failure correlate with published expression signatures know to predict for distant metastases [22, 23].

The use of novel imaging techniques has also improved the ability to document oligometastatic disease. While no consensus definition of oligometastatic disease for prostate cancer exists, ≤ 3 up to 5 metastatic sites has often been applied as the cut off in clinical trials of focal radiation therapy [24–27]. Therefore, the current study suggests that a substantial proportion of patients (10 of 32 patients in this series) could be considered for enrollment on clinical trials for ablative therapy to oligometastases.

The authors acknowledge the limitations of this study, which include it being a retrospective secondary analysis of two different PSMA PET agents, small sample size, and the lack of confirmatory biopsy on majority of prostate fossa lesions. Regarding confirmatory biopsies, one significant limitation for small lesions seen on mpMRI and ^18^F-PSMA imaging is the lack of ultrasound correlate, which makes accurately targeting lesions for ultrasound guided biopsy difficult [28]. In this study every effort was made, particularly in the cases of prostate fossa recurrence, to obtain complete radiation records and confirm the dose received to each lesion. However, due to the time interval between radiation and PSMA PET, electronic plans were not available for all patients and cognitive fusion was required based on anatomic landmarks.

## Conclusions

The current study reports that distant recurrence remains the predominant pattern of failure in patients who experience biochemical recurrence after radical prostatectomy and post-operative radiotherapy to the prostate fossa with or without elective nodal irradiation. In the current study, approximately 1 in every 5 patients with unremarkable conventional imaging findings have detectable in-field failure despite use of post-operative radiotherapy, and 1 in 3 have oligometastatic disease. This emphasizes the importance of future research in tailoring radiotherapy target delineation, investigating radiation dose escalation, or use of systemic treatment concomitantly with radiotherapy to optimize local control and improve metastasis free survival in these patients.

## Supplementary Information

**Additional file 1: Table 1.** Summary showing univariate multinomial logistic regression for each predictor variable computed for each model indicated under the column “Intercept” based on site of recurrence.

**Additional file 2: Table 2.** Summary information for clinical features for each of the sites of recurrence of a multinomial model.

**Additional file 3: Table 3.** Multinomial multivariate associations of significant clinical variables from univariate analysis with site of recurrence at ^18^F-PSMA-PET/CT.

## Data Availability

The datasets supporting the conclusions of this article are included within the article and supplementary material.

## Abbreviations

AIC: Akaike Information Criterion
ADT: Androgen deprivation therapy
CT: Computerized tomography
DCFBC: N-[N-[(S)-1,3-dicarboxypropyl]carbamoyl]-4-F-fluorobenzyl-l-cysteine
DCFPyL: 2-(3-{1-Carboxy-5-[(6-[^18^F]fluoro-pyridine-3-carbonyl)-amino]-pentyl}-ureido)-pentanedioic acid
IMRT: Intensity-modulated radiation therapy
MRI: Magnetic resonance imaging
mpMRI: Multiparametric magnetic resonance imaging
NCCN: National Comprehesive Cancer Network
PCa: Prostate cancer
PET: Positron emission tomography
PSA: Prostate-specific antigen
PSMA: Prostate Membrane Specific Antigen
PORT: Post-operative radiation therapy
