# Supplementary material for: Pattern of failure in prostate cancer previously treated with radical prostatectomy and post-operative radiotherapy: a secondary analysis of two prospective studies using novel molecular imaging techniques
Source: Radiat Oncol. 2021 Feb 10;16:32. doi: 10.1186/s13014-020-01733-x (PMC7874470; doi:10.1186/s13014-020-01733-x)
Supplement: Supplementary file 1 — Additional file 1: Table 1. Summary showing univariate multinomial logistic regression for each predictor variable computed for each model indicated under the column “Intercept” based on site of recurrence. [file 13014_2020_1733_MOESM1_ESM.docx]

Table 1. Summary showing univariate multinomial logistic regression for each predictor variable computed for each model indicated under the column “Intercept” based on site of recurrence.

|  |  |  |  |  |  |  |  | Intercept | | Goodness of fit | |  |
| --- | --- | --- | --- | --- | --- | --- | --- | --- | --- | --- | --- | --- |
|  | Coefficient (β)^a^ | Std. Error^b^ | t value | p value | OR | 2.50% CI | 97.50% CI | M\|LN | LN\|LR | Residual deviance | AIC |  |
| Age at diagnosis | -0.08 | 0.06 | -1.25 | 0.21 | 0.93 | 0.82 | 1.04 | -4.3 | -3.1 | 63.30 | 69.20 |  |
| Gleason Grade Group 1 | ref |  |  |  |  |  |  | -17.46 | -16.04 | 56.20 | 68.20 |  |
| Gleason Grade Group 2 | -17.03 | 0.59 | -28.81 | 0.00*** | 0.00 | 0.00 | 0.00 |  |  |  |  |  |
| Gleason Grade Group 3 | -18.30 | 0.61 | -29.99 | 0.00*** | 0.00 | 0.00 | 0.00 |  |  |  |  |  |
| Gleason Grade Group 4 | -17.97 | 0.63 | -28.53 | 0.00*** | 0.00 | 0.00 | 0.00 |  |  |  |  |  |
| Gleason Grade Group 5 | -17.36 | 0.81 | -21.54 | 0.00*** | 0.00 | 0.00 | 0.00 |  |  |  |  |  |
| Intermediate Risk | ref |  |  |  |  |  |  | -0.84 | 0.45 | 60.54 | 66.54 |  |
| High Risk | -1.50 | 0.73 | -2.06 | 0.04** | 0.22 | 0.05 | 0.93 |  |  |  |  |  |
| Margins Negative | ref |  |  |  |  |  |  | 0.12 | 1.27 | 64.96 | 70.96 |  |
| Margins Positive | 0.00 | 0.69 | 0.00 | 1.00 | 1.00 | 0.26 | 3.86 |  |  |  |  |  |
| SVI Negative | ref |  |  |  |  |  |  | -0.23 | 0.99 | 61.9 | 67.90 |  |
| SVI Positive | -1.44 | 0.89 | -1.61 | 0.11 | 0.24 | 0.04 | 1.36 |  |  |  |  |  |
| ECE Negative | ref |  |  |  |  |  |  | -0.43 | 0.77 | 63.01 | 69.01 |  |
| ECE Positive | -0.96 | 0.70 | -1.38 | 0.17 | 0.38 | 0.10 | 1.49 |  |  |  |  |  |
| Node Negative (N0) | ref |  |  |  |  |  |  | 16.55 | 17.72 | 63.53 | 71.53 |  |
| N1 | 16.54 | 0.39 | 42.53 | 0.00*** | 15307970.00 | 7141870.00 | 32811270.00 |  |  |  |  | # |
| Nx | 16.19 | 0.61 | 26.70 | 0.00*** | 10795880.00 | 3287769.00 | 35449860.00 |  |  |  |  | # |
| Post-operative PSA | 0.43 | 0.76 | 0.57 | 0.57 | 1.54 | 0.35 | 6.80 | 0.20 | 1.36 | 64.64 | 70.64 |  |
| No prior ADT | ref |  |  |  |  |  |  | 0.19 | 1.23 | 62.09 | 68.09 |  |
| Prior ADT | 0.00 | 0.69 | 0.00 | 1.00 | 1.00 | 0.26 | 3.87 |  |  |  |  |  |
| PSA at Recurrence | 1.21 | 0.95 | 1.27 | 0.20 | 3.36 | 0.52 | 21.61 | 0.61 | 1.82 | 63.18 | 69.18 |  |
| Time to Failure | 0.00 | 0.01 | 0.18 | 0.86 | 1.00 | 0.98 | 1.02 | 0.19 | 1.34 | 64.93 | 70.93 |  |
| Radiation Dose | 0.12 | 0.18 | 0.66 | 0.51 | 1.13 | 0.79 | 1.60 | 8.33 | 9.29 | 57.39 | 63.39 |  |
| PSA at PSMA PET | -0.40 | 0.18 | -2.25 | 0.02** | 0.67 | 0.48 | 0.95 | -1.21 | 19.00 | 55.36 | 61.36 |  |

^a^Regression coefficient; ^b^Standard error of β; *p<0.1; **p<0.05; ***p<0.01; #values flagged for large OR and CI
