# Supplementary material for: Pattern of failure in prostate cancer previously treated with radical prostatectomy and post-operative radiotherapy: a secondary analysis of two prospective studies using novel molecular imaging techniques
Source: Radiat Oncol. 2021 Feb 10;16:32. doi: 10.1186/s13014-020-01733-x (PMC7874470; doi:10.1186/s13014-020-01733-x)
Supplement: Supplementary file 2 — Additional file 2: Table 2. Summary information for clinical features for each of the sites of recurrence of a multinomial model. [file 13014_2020_1733_MOESM2_ESM.docx]

Table 2. Summary information for clinical features for each of the sites of recurrence of a multinomial model.

| Stage | Clinical feature | Coefficient (β)^a^ | Std. error^b^ | t value | p value | 2.5% CI | 97.5% CI |
| --- | --- | --- | --- | --- | --- | --- | --- |
| M | (Intercept) | 1.81 | 0.60 | 3.02 | 0.01*** | 0.57 | 3.06 |
|  | Gleason Grade Group 2 | -0.32 | 0.37 | -0.88 | 0.39 | -1.09 | 0.44 |
|  | Gleason Grade Group 3 | -0.70 | 0.42 | -1.68 | 0.11 | -1.56 | 0.16 |
|  | Gleason Grade Group 4 | -0.60 | 0.47 | -1.27 | 0.22 | -1.57 | 0.37 |
|  | Gleason Grade Group 5 | -0.46 | 0.52 | -0.87 | 0.39 | -1.53 | 0.62 |
|  | High Risk | -0.04 | 0.29 | -0.14 | 0.89 | -0.63 | 0.55 |
|  | N0 | 0.43 | 0.50 | 0.86 | 0.40 | -0.60 | 1.46 |
|  | N1 | 0.12 | 0.55 | 0.22 | 0.83 | -1.02 | 1.27 |
|  | PSA at PSMA-PET | -0.04 | 0.02 | -2.17 | 0.04** | -0.08 | 0.00 |
| LN | (Intercept) | 2.12 | 0.58 | 3.63 | 0.00*** | 0.91 | 3.33 |
|  | Gleason Grade Group 2 | -0.40 | 0.36 | -1.12 | 0.27 | -1.15 | 0.34 |
|  | Gleason Grade Group 3 | -0.07 | 0.40 | -0.19 | 0.85 | -0.91 | 0.76 |
|  | Gleason Grade Group 4 | -0.22 | 0.46 | -0.48 | 0.63 | -1.16 | 0.72 |
|  | Gleason Grade Group 5 | -0.56 | 0.51 | -1.10 | 0.28 | -1.61 | 0.49 |
|  | High Risk | -0.09 | 0.28 | -0.31 | 0.76 | -0.66 | 0.49 |
|  | N0 | -0.19 | 0.48 | -0.39 | 0.70 | -1.19 | 0.81 |
|  | N1 | -0.15 | 0.54 | -0.28 | 0.78 | -1.26 | 0.96 |
|  | PSA at PSMA-PET | 0.02 | 0.02 | 1.18 | 0.25 | -0.02 | 0.06 |
| LR | (Intercept) | 1.06 | 0.49 | 2.15 | 0.04** | 0.04 | 2.09 |
|  | Gleason Grade Group 2 | 0.73 | 0.30 | 2.40 | 0.03** | 0.10 | 1.36 |
|  | Gleason Grade Group 3 | 0.77 | 0.34 | 2.26 | 0.03** | 0.07 | 1.48 |
|  | Gleason Grade Group 4 | 0.82 | 0.39 | 2.11 | 0.05* | 0.02 | 1.62 |
|  | Gleason Grade Group 5 | 1.01 | 0.43 | 2.36 | 0.03** | 0.12 | 1.90 |
|  | High Risk | 0.12 | 0.24 | 0.53 | 0.60 | -0.36 | 0.61 |
|  | N0 | -0.24 | 0.41 | -0.59 | 0.56 | -1.09 | 0.61 |
|  | N1 | 0.03 | 0.46 | 0.06 | 0.95 | -0.92 | 0.97 |
|  | PSA at PSMA-PET | 0.02 | 0.02 | 1.24 | 0.23 | -0.01 | 0.05 |

^a^Regression Coefficient; ^b^Standard error of β; M = metastatic disease; LN = Regional lymph nodes; LR = Local recurrence; *p<0.1; **p<0.05; ***p<0.01
