# Supplementary material for: Pattern of failure in prostate cancer previously treated with radical prostatectomy and post-operative radiotherapy: a secondary analysis of two prospective studies using novel molecular imaging techniques
Source: Radiat Oncol. 2021 Feb 10;16:32. doi: 10.1186/s13014-020-01733-x (PMC7874470; doi:10.1186/s13014-020-01733-x)
Supplement: Supplementary file 3 — Additional file 3: Table 3. Multinomial multivariate associations of significant clinical variables from univariate analysis with site of recurrence at 18F-PSMA-PET/CT. [file 13014_2020_1733_MOESM3_ESM.docx]

Table 3: Multinomial multivariate associations of significant clinical variables from univariate analysis with site of recurrence at ^18^F-PSMA-PET/CT.

|  | Coefficient (β)^a^ | Std. Error^b^ | t value | p value | OR | 2.50% CI | 97.50% CI |  |
| --- | --- | --- | --- | --- | --- | --- | --- | --- |
| Gleason Grade Group 1 | ref |  |  |  |  |  |  |  |
| Gleason Grade Group 2 | -18.55 | 1.29 | -14.41 | 0.00*** | 0.00 | 0.00 | 0.00 |  |
| Gleason Grade Group 3 | -22.35 | 0.80 | -27.85 | 0.00*** | 0.00 | 0.00 | 0.00 |  |
| Gleason Grade Group 4 | -21.95 | 0.97 | -22.58 | 0.00*** | 0.00 | 0.00 | 0.00 |  |
| Gleason Grade Group 5 | -22.11 | 1.12 | -19.80 | 0.00*** | 0.00 | 0.00 | 0.00 |  |
| Intermediate Risk | ref |  |  |  |  |  |  |  |
| High Risk | 0.73 | 1.49 | 0.49 | 0.63 | 2.07 | 0.11 | 38.50 |  |
| Node Negative (N0) | ref |  |  |  |  |  |  |  |
| N1 | 18.02 | 0.87 | 20.74 | 0.00*** | 66725840.00 | 12155330.00 | 366287000.00 | # |
| Nx | 14.55 | 1.17 | 12.45 | 0.00*** | 2091235.00 | 211398.70 | 20687280.00 | # |
| PSA at PSMA PET | -0.71 | 0.29 | -2.44 | 0.01*** | 0.49 | 0.28 | 0.87 |  |
|  | | | | | | | | |
| Intercept | M\|LN | -5.08 | 0.68 | -7.48 | 0.00 |  |  |  |
| Intercept | LN\|LR | -2.97 | 0.86 | -3.45 | 0.00 |  |  |  |
| Goodness of fit | |  |  |  |  |  |  |  |
|  | Residual Deviance | 39.74 |  |  |  |  |  |  |
|  | AIC | 59.74 |  |  |  |  |  |  |

^a^Regression Coefficient; ^b^Standard error of β*p<0.1; **p<0.05; ***p<0.01; #Flagged due to high OR values with large CI
